# Coevaporation Stabilizes Tin-Based Perovskites in a Single Sn-Oxidation State

**DOI:** 10.1021/acs.nanolett.2c02204

**Published:** 2022-08-23

**Authors:** Ajay Singh, Jeremy Hieulle, Joana Ferreira Machado, Sevan Gharabeiki, Weiwei Zuo, Muhammad Uzair Farooq, Himanshu Phirke, Michael Saliba, Alex Redinger

**Affiliations:** † Department of Physics and Materials Science, University of Luxembourg, Luxembourg City L-1511, Luxembourg; ‡ Institute for Photovoltaics (IPV), University of Stuttgart, Pfaffenwaldring 47, 70569 Stuttgart, Germany; @ Helmholtz Young Investigator Group FRONTRUNNER, IEK5-Photovoltaik, Forschungszentrum Jülich, 52425, Jülich, Germany

**Keywords:** Tin-perovskites, Sn[2+] oxidation, photostability, lead-free perovskite solar cells, open-cricuit voltage

## Abstract

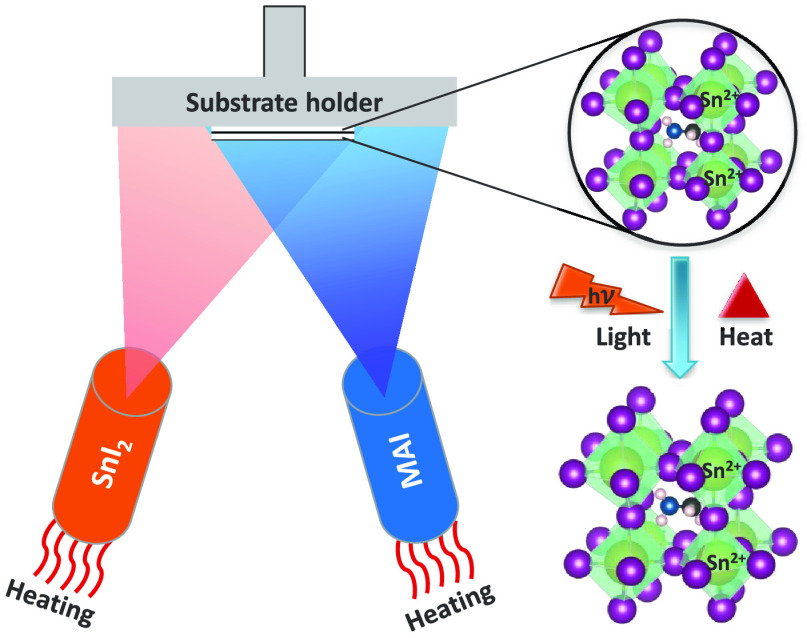

Chemically processed methylammonium tin-triiodide (CH_3_NH_3_SnI_3_) films include Sn in different oxidation states, leading to poor stability and low power conversion efficiency of the resulting solar cells (PSCs). The development of absorbers with Sn [2+] only has been identified as one of the critical steps to develop all Sn-based devices. Here, we report on coevaporation of CH_3_NH_3_I and SnI_2_ to obtain absorbers with Sn being only in the preferred oxidation state [+2] as confirmed by X-ray photoelectron spectroscopy. The Sn [4+]-free absorbers exhibit smooth highly crystalline surfaces and photoluminescence measurements corroborating their excellent optoelectronic properties. The films show very good stability under heat and light. Photoluminescence quantum yields up to 4 × 10^–3^ translate in a quasi Fermi-level splittings exceeding 850 meV under one sun equivalent conditions showing high promise in developing lead-free, high efficiency, and stable PSCs.

The rapid advancement in the development of hybrid organic inorganic perovskite photovoltaics during the last years led to high power conversion efficiencies (PCE), breaking the 25% benchmark for small area devices.^1^ However, their large scale commercialization is hampered by the toxicity of lead combined with an insufficient long-term stability.^2,3^ Independent of the current regulations, highly efficient Pb-free solar cells would be highly preferable, much safer, and would trigger additional investments in this emerging technology. Substituting lead with less toxic metals has been a scientific challenge and possible candidates to replace Pb are Ge, Sn, and Sb, where tin (Sn) is currently regarded as the best replacement.^4−8^ Reports have suggested that methylammonium tin triiodide (CH_3_NH_3_SnI_3_, hereafter called MASI) exhibits favorable optoelectronic properties such as a direct bandgap of (1.15–1.35 eV), a high absorption coefficient (∼2 × 10^4^ cm^–1^), high charge carrier mobilities (1–2000 cm^2^ V^–1^ S^–1^), and long carrier diffusion lengths (0.1–1 μm).^5,9−12^ However, tin-based hybrid perovskites, including MASI, often undergo a decoloration within minutes of exposure to ambient conditions, suggesting a rapid degradation.^5,11,13^ Moreover, changes in oxidation state of the Sn (Sn [2+] →Sn [4+]) leads to p-type doping of the perovskite absorber, known as “self-doping”, which is accompanied by a strong increase in charge carrier recombination rates and with a reduction in diffusion length.^5,11−13^ Consequently, tin-based solution-processed photovoltaic devices result in poor stability and low power conversion efficiency as compared to lead-based devices.^5,13−19^

Both the lower PCE and the instability have been attributed to the high density of intrinsic defects such as Sn vacancies and oxidized species (Sn [4+]) in the absorber.^5,13,20,21^ It is therefore of paramount importance to address the issue of the different Sn oxidation states. Strategies to improve solution-based absorber properties include solvent engineering, use of additives, pre- and post-treatments, doping, employing buffer layers, and many other chemical processes.^10,14,22,23^ However, all these processes use additional chemical and/or physical processes, which might further trigger the formation of Sn [4+].^24^

In this study, we show how the oxidation of Sn [2+] to Sn [4+] can be prevented and how this impacts the absorber stability. The fabrication method has been physical vapor deposition (PVD), which is a well-known solvent free synthesis route to obtain smooth and homogeneous films without pinholes.^25−27^ The MASI perovskite films synthesized in this study were obtained by coevaporation of methylammonium iodide (MAI) and tin di-iodide (SnI_2_) on top of ITO substrates. By controlling the MAI source temperature, stoichiometric MASI films as well as MASI films with excess SnI_2_ (hereafter called nonstoichiometric films) could be synthesized. More experimental details can be found in the Supporting Information (SI). In this Letter, we focus on stoichiometric films only.

The synthesized films were characterized with X-ray diffraction (XRD) to confirm the crystalline structure and the phase purity. The chemical composition was analyzed by X-ray photoelectron spectroscopy (XPS). The surface properties were analyzed by Kelvin probe force microscopy measurements carried out under ultrahigh vacuum (UHV) conditions. Finally, the optoelectronic properties of the coevaporated films were studied via photoluminescence quantum yield and with quasi Fermi-level splitting measurements at one sun equivalent conditions. Stability against light and heat was investigated via photoluminescence (PL) and XRD measurements. Our results show that the absence of Sn [4+] leads to absorbers that are stable under continuous light illumination for several days, which is a prerequisite for future high-performance devices.

Figure 1a depicts a typical XRD diffractogram of a stoichiometric MASI film grown via PVD. The diffraction peaks could be assigned to a polycrystalline pseudocubic phase in agreement with literature.^5,28,29^ For the MASI films with excess SnI_2_, along with the MASI peaks, SnI_2_ peaks were observed, as shown in Figure S1. We note that the texture of the films is strongly dependent on the growth conditions. More details on the growth process itself will be discussed elsewhere. In the present case, the absorbers all had very similar XRD diffractograms.

**Figure 1:**
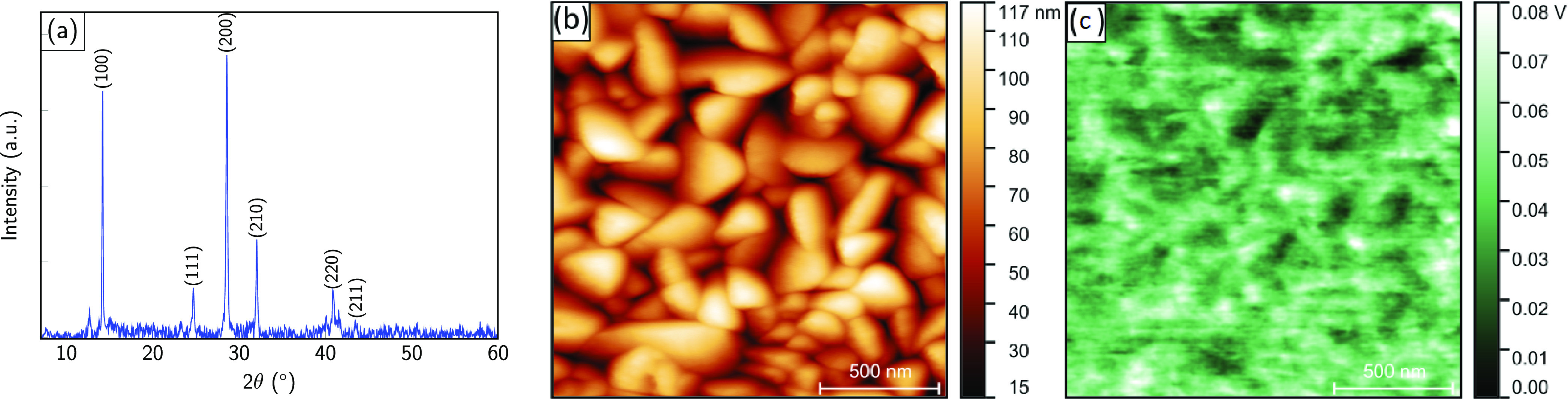
(a) X-ray diffractogram, (b) topographic image measured via AFM, and (c) contact potential difference map of the PVD-grown MASI perovskite films. (b,c) Images acquired via single pass frequency modulation AFM/KPFM.

Figure 1b,c depicts atomic force microscopy (AFM) and Kelvin probe force microscopy (KPFM) measurements for stoichiometric MASI films carried out in ultrahigh vacuum. The transfer from the nitrogen filled glovebox to the UHV chamber was carried out without air exposure. We do see grains with an average size of approximately 200 nm. In KPFM, we measure the contact potential difference between the tip and the sample with nanometer resolution. We observed variations of only 80 meV, with no obvious secondary phases and no visible contrast at the grain boundaries. The contrast visible in Figure 1c arises almost exclusively from different facets of the grains, which is a direct consequence of different surface terminations and surface dipoles. However, the magnitude of the variations is much smaller as compared to those observed previously on CH_3_NH_3_PbI_3_.^30^ We noted a slight variation of the work function with time, which might be an indication of light-induced changes due to stray light of the cantilever setup. These light-induced changes will be discussed further in the following. Furthermore, we recorded top-view scanning electron microscopy (SEM) images of the PVD-grown MASI film as shown in Figure S2. The SEM image shows a very well patterned, smooth crystalline film supporting the XRD, AFM, and KPFM results. Large area KPFM and AFM measurements for nonstoichiometric MASI film in N_2_ environment are shown in Figure S3.

The surface composition and the amount of Sn [4+] was determined via X-ray photoelectron spectroscopy (XPS) measurements. In analogy to the KPFM measurements, this analysis was carried out without exposure to ambient conditions. In total, four different samples were analyzed and the results are summarized in Figure 2. We focus on the Sn(3d) core levels here since they can be used to determine the oxidation state of the Sn. The top graph depicts the result from the coevaporated film (denoted as PVD grown MASI film), followed by a comparison with a state of the art solution-based formamidinium tin triiodide film (denoted as FASI).

**Figure 2:**
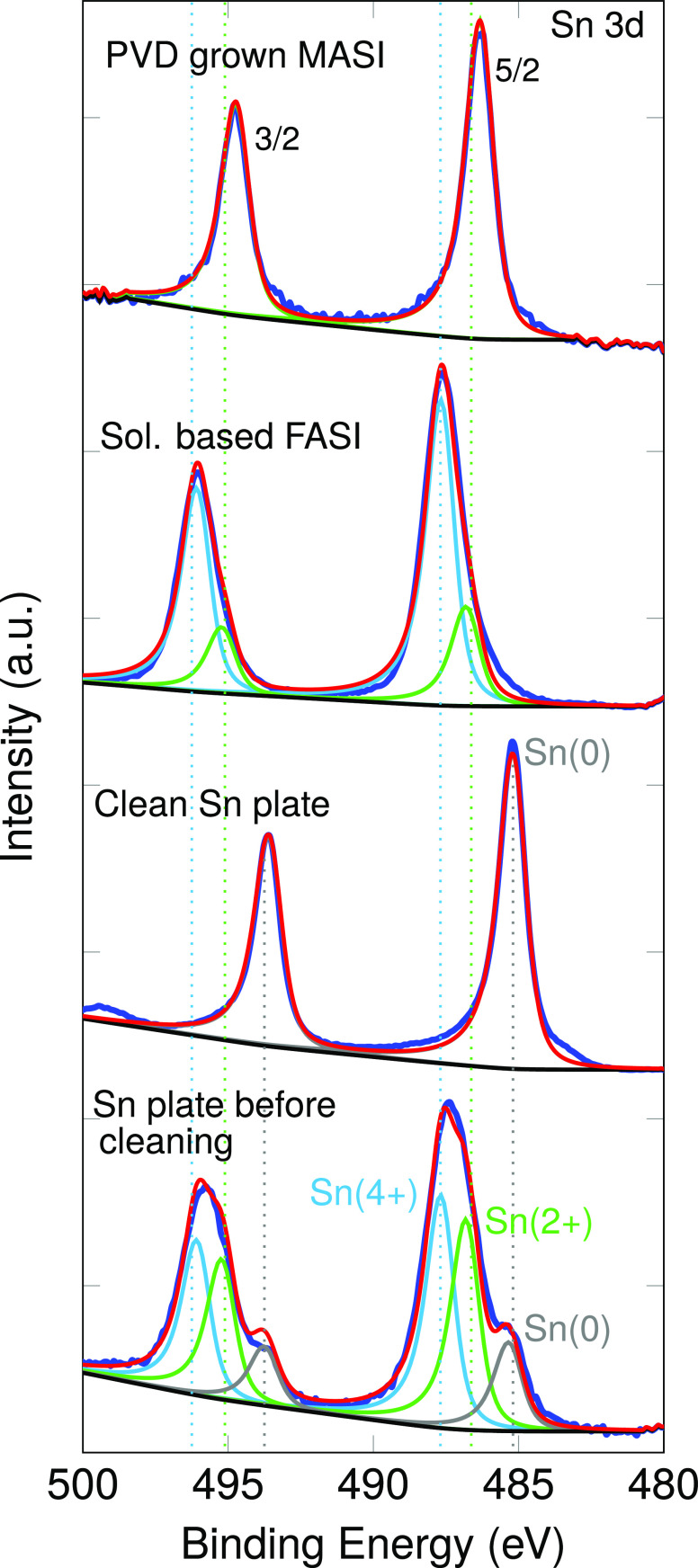
X-ray photoelectron spectroscopy curves of a PVD grown MASI sample and comparison with other reference samples. The blue curves depict the measured spectra whereas the red one is a fit to the data. From top to bottom: PVD grown MASI sample; solution-based FASI sample; Sn plate cleaned in ultrahigh vacuum by Ar^+^ sputtering; oxidized Sn plate before sputtering. The distinct oxidation states are labeled as follows Sn [0] (gray color), Sn [2+] (green color), and Sn [4+] (light blue color). The oxidized Sn plate and the solution-based FASI samples exhibit two common oxidation states (Sn [2+], Sn [4+]), while the PVD grown MASI perovskite possess a single oxidation state, namely Sn [2+].

The spectrum measured on the PVD grown sample is composed of a typical doublet peak associated with the expected spin–orbit-coupling (SOC) split peaks (*J*_tot_ = 5/2; 3/2) of the Sn(3d) state. The curve can be well fitted using a Voight function lineshape with only two components, one for each SOC peak at 486.4 and 494.8 eV, respectively. However, fitting without detailed knowledge of the lineshape is imprecise and small amounts of a different oxidation state are easily obscured.

Therefore, we measured the XPS spectrum of a 99.99% pure Sn plate cleaned in vacuum by Ar^+^ sputtering (depicted in Figure 2, third row from top). We observed the expected SOC doublet peak with a sharp single component. The full width at half-maximum (fwhm) obtained after fitting this curve is of 1.1 eV and corresponds to a pure state with a single degree of oxidation, that is, Sn [0]. This fwhm needs to be used as an input parameter for the subsequently fit of the PVD-grown sample. This is a well-justified procedure since the shape and the experimental broadening arise from the characteristic of the device used, as well as the photoemission process of the Sn(3d) element. This procedure allows us to make sure that the spectrum of the PVD-grown film is fitted with the correct lineshape and thereby with high accuracy. The result of this fit is depicted in the figure as a solid red line. On a side note, it was found that the binding energy positions of the zero-oxidation state Sn [0] doublet are 485.3 and 493.7 eV. These values are much lower than the components measured for the PVD-grown sample.

In order to make an unambiguous identification of the correct Sn oxidation state we measured the same Sn plate before sputtering, which gives us some precious information on the energy position of the higher oxidation states of the Sn(3d) core level. As shown in Figure 2 (bottom), we obtained three SOC doublets with one of them at the positions of the Sn [0] oxidation state, and two other ones at higher binding energies (486.7 eV; 495.1 and 487.7 eV; 496.1 eV). It is important to note that higher binding energies usually correspond to a higher degree of oxidation.^31^ In addition, the most commonly observed degree of oxidation in the literature for Sn are [2+] and [4+].^32^ Therefore, we can associate the doublet at 486.7 eV, 495.1 eV to a Sn [2+] degree of oxidation, and the doublet at 487.7 eV, 496.1 eV corresponds to the Sn [4+] degree of oxidation.

Hence, the Sn-plate allowed us to determine the correct lineshape and the position of the core levels for the different oxidation states. Using these values as input parameters allowed us to fit the PVD-grown MASI with a single doublet, which corresponds to a single oxidation state, namely Sn [2+]. The surface elemental composition of the film determined by the XPS is MA_0.84_SnI_3_, which is close to the stoichiometric MA_1.0_Sn_1.0_I_3.0_ composition and within the experimental error of XPS.

For the solution-based FASI sample, the XPS spectrum cannot be fitted with only one doublet, as it was done for the PVD-grown MASI sample. Instead, four Voight functions (2 doublets) needed to be taken into account (Figure 2, second row from top). The fit was done with the exact same lineshape and with the same fwhm as the PVD sample. It is found that one doublet has very similar bindings energies as the one measured for the PVD grown sample (e.g., 486.7 and 495.1 eV) and might be related to the same oxidation state of the Sn(3d) element, namely Sn [2+]. The slight shift toward higher binding energy can be understood from the slight change in chemical environment due to the use of the formamidinium cation in the solution-based sample with respect to the methylammonium cation used in the PVD sample. In addition to those peaks, a second doublet with a maximum intensity at the binding energies of 487.7 and 496.1 eV was identified, which is related to a higher degree of oxidation of Sn(3d), namely Sn [4+].

In tin-based perovskite solar cells, the presence of Sn [4+] oxidation state has been shown to be detrimental to device performance and stability.^13,14,20,21^ Therefore, the absence of Sn [4+] in the PVD-grown MASI sample opens a great opportunity for realizing stable Pb-free perovskite devices. However, it still needs to be determined if the samples are indeed stable and of high optoelectronic quality.

To get insight into the photostability of the PVD-grown films, photoluminescence quantum yield measurements (*Q*_e_^lum^) were performed in two different setups and different environments. *Q*_e_^lum^ is defined as the ratio of the emitted and the impinging photon flux and directly relates to the quasi Fermi-level splitting μ via^33^
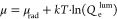
1The ideal case, which corresponds to radiative recombination only is given by μ_rad_ and is equal to the Shockley–Queisser *V*_OC_ in the case of a step like absorption.^34^ Because μ corresponds to the upper limit of the *V*_OC_ in the final device, the extraction of *Q*_e_^lum^ directly reflects the potential of the Pb-free absorbers in solar cell devices. The photostability in a nitrogen environment is depicted in Figure 3a. The temporal evolution of *Q*_e_^lum^ under continuous illumination was measured in a photoluminescence imaging setup, which allows one to measure laterally resolved PL images with micrometer resolution. For the following discussion we assume a bandgap of *E*_g_ = 1.28 eV, which corresponds to the PL peak position, prior to illumination (details see later).

**Figure 3:**
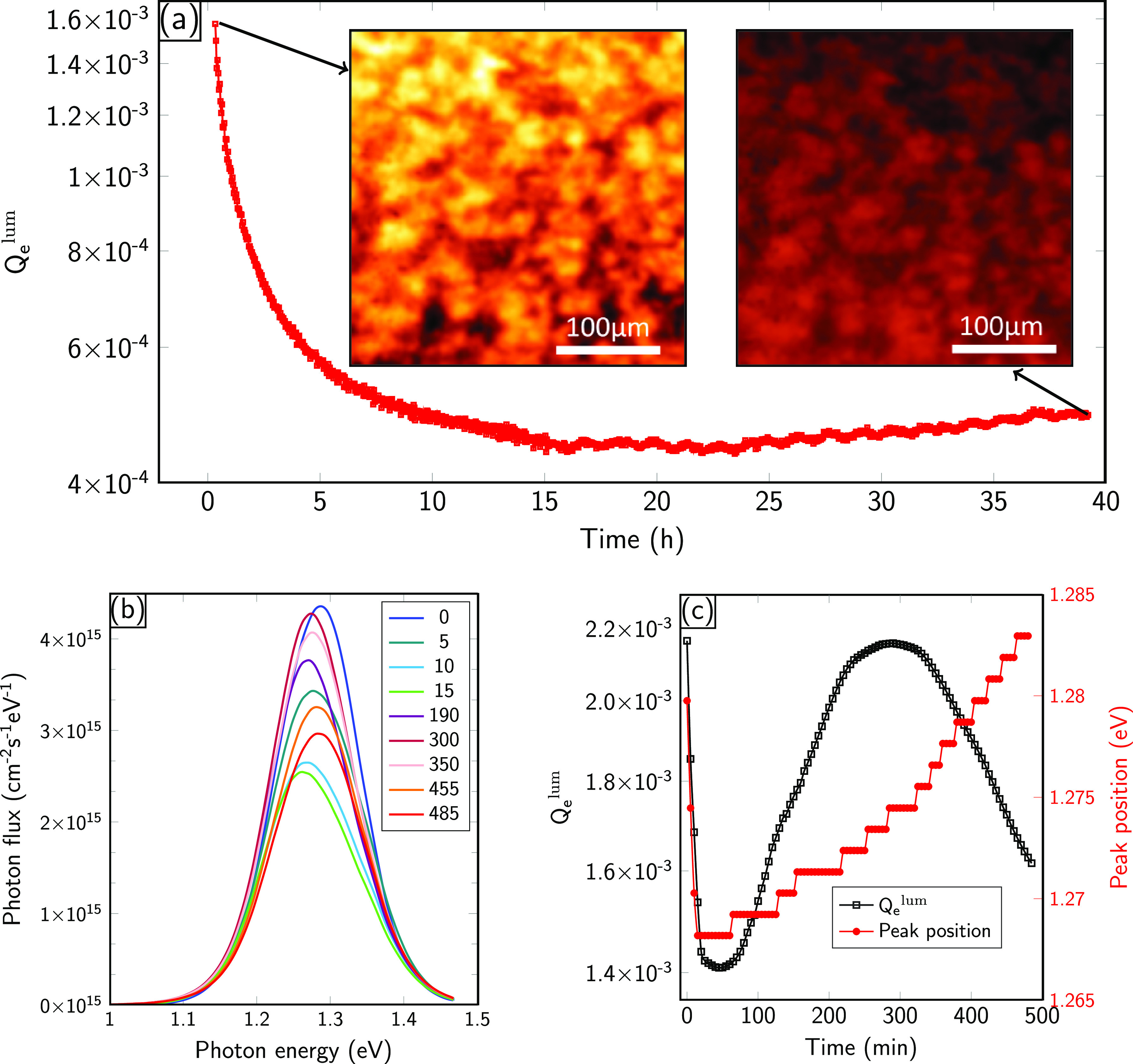
(a) PLQY (*Q*_e_^lum^) over time extracted from PL images of the MASI films in N_2_ ambient. The PL images are recorded by continuously illuminating the sample with pulsed green laser running at energy fluence of 60 m W cm^–2^. (b) The PL spectrum recorded at different intervals between 0 and 485 min in the air while continuously illuminating the sample with a red laser of 1 sun equivalent injection. (c) PLQY and the shift in PL peak position extracted from the measurements in (b).

The impinging photon flux was set to 60 mW/cm^2^, which corresponds to 0.84 sun equivalents, assuming *E*_g_ = 1.28 eV. The initial *Q*_e_^lum^ value of 1.6 × 10^–3^ corresponds to a loss of 163 meV compared to μ_rad_. This translates to a quasi Fermi level splitting of μ = 867 meV for the used value of *E*_g_. This highlights that the as-grown absorbers were of excellent optoelectronic quality. After continuous illumination of approximately 40 h, we observed a decrease in *Q*_e_^lum^ by only a factor of 3, which corresponds to an additional loss of only 29 meV. The collected images show a homogeneous drop of the PL yield over the complete surface as exemplified by the two insets in Figure 3a. We note however that we do observe lateral variations (much larger than the typical grain size) in the PL yield and consequently also in *Q*_e_^lum^. The variations can either arise from differences in the number of nonradiative recombination centers or due to slight changes in PL peak position.

In order to track the changes in PL peak position, we carried out spectrally resolved measurements in a confocal setup (in air) and the results are presented in Figure 3b,c. The measurements were carried out for 485 min continuous illumination, and spectra were recorded every 5 min. A subset of the data set is shown in Figure 3b whereas *Q*_e_^lum^ and the PL peak position are depicted in Figure 3c. The initial values of *Q*_e_^lum^ are in excellent agreement with the previous measurements, although the setups were calibrated independently. The slight difference might be due to sample to sample variations or due to the different atmosphere. We do see a sharp decrease in *Q*_e_^lum^ by roughly a factor of 2 in the first 15 min followed by partial recovery up to 300 min. The PL peak position exhibits similar variations with an initial decrease by roughly 10 meV followed by a recovery. The steplike changes in peak position are attributed to the resolution of the used monochromator. Comparing the measurements in air and in N_2_ allows us to conclude that the absorbers exhibit an excellent stability against continuous light illumination, independent of the surrounding atmosphere. The subtle changes in PL peak position and the discrepancy between the temporal evolution of *Q*_e_^lum^ are currently not clear and a dedicated study is necessary. At present, we attribute the changes to a partial substitution of iodine with oxygen in the near surface region as measured with XPS after the light exposure (not shown here).

To get insight into the thermal stability of the PVD-grown perovskite films, a MASI film grown on glass was subjected to 60 °C on a hot plate inside a glovebox (N_2_ environment). N_2_ was chosen since the solar cell devices are usually encapsulated, rendering the oxygen and moisture levels very low. The temperature of 60 °C corresponds to the equilibrium surface temperature of the perovskite under continuous one sun illumination without cooling in a laboratory environment of around 25 °C. The chosen temperature can therefore be considered as the minimum temperature a device needs to sustain under normal operation conditions.

XRD spectra were recorded at several time intervals by taking the sample off the hot plate while maintaining the N_2_ environment. Repeated cycles of sample annealing, followed by XRD analysis were performed, as depicted in Figure 4. The diffractograms are vertically shifted to improve the visibility. The results clearly showed that, after 22 h of heating at 60 °C followed by 2 h of heating at 70 °C, there was no significant change in the bulk crystal structure of the MASI films. The intensity of all the peaks stayed constant and no additional peaks could be observed. This suggested that the films were highly stable against temperature changes and should not undergo degradation upon sunlight heating. However, care needs to be taken to keep the sample in an oxygen-free environment and the extraction layers need to be chosen such that they do not deteriorate the absorber stability.

**Figure 4:**
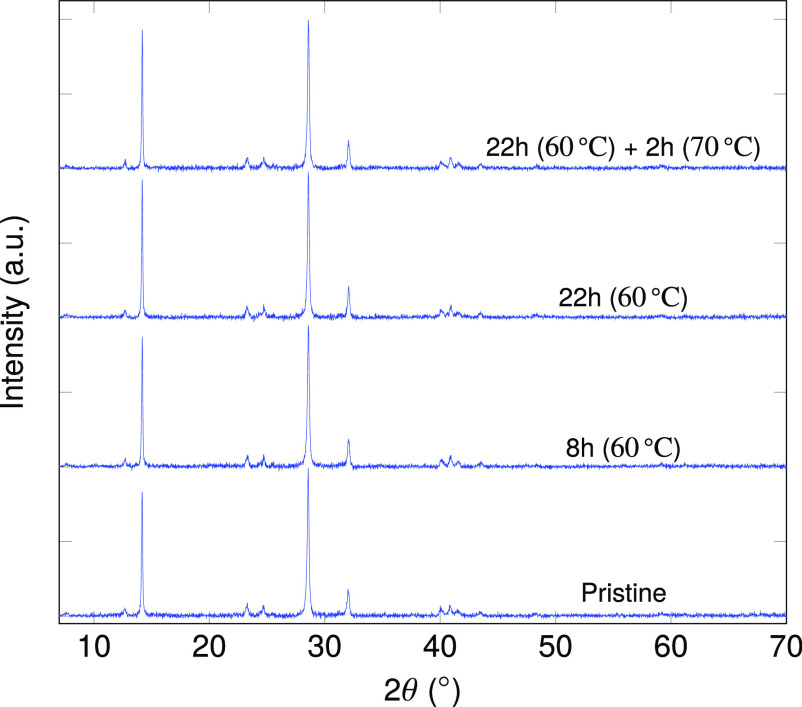
X-ray diffraction measurement for pristine (*t* = 0) and heated MASI film. The film is heated at 60 °C for 22 h, and the XRD spectra were recorded after the indicated time intervals. The temperature is then increased to 70 °C and the heating is done for 2 additional hours (22 h +2 h). No obvious change is observed in the XRD spectra upon heating.

In summary, we have shown that coevaporation of SnI_2_ and CH_3_NH_3_I allows one to synthesize MASI perovskite films without detrimental Sn in the Sn [4+] oxidation state. This also yields very homogeneous surfaces as demonstrated by AFM and KPFM, not only in terms of roughness but also in terms of work function. The homogeneous workfunction and the absence of preferential contrast at the grain boundaries are highly beneficial for devices. In addition, we measured high *Q*_e_^lum^ values exceeding 1 × 10^–3^, which translates into quasi Fermi-levels splitting well beyond 850 meV. We emphasize that these estimated open circuit voltages are high enough to reach power conversion efficiencies in the range of 20%, assuming that suitable extraction layers can be identified that do not deteriorate the interface quality. The bare absorbers showed an excellent stability against continuous light exposure and heat, which are prerequisites for stable devices. More work is necessary to disentangle the changes in PL peak position and *Q*_e_^lum^. This work shows that the amount of Sn [4+] can be reduced drastically if the growth processes are carried without solvents and in an oxygen free atmosphere. This opens the door to develop high-performance low-bandgap perovskites, which have ideal bandgaps for single and multijunction devices.

## Supporting Information Available

Experimental section, XRD of SnI_2_ rich MASI film, SEM image of pristine MASI film, large area AFM/KPFM in N_2_, PL degradation (PDF)
