# Supplementary material for: Coevaporation Stabilizes Tin-Based Perovskites in a Single Sn-Oxidation State
Source: Nano Lett. 2022 Aug 23;22(17):7112–8. doi: 10.1021/acs.nanolett.2c02204 (PMC9479155; doi:10.1021/acs.nanolett.2c02204)
Supplement: Supplementary file 1 — nl2c02204_si_001.pdf [file nl2c02204_si_001.pdf]

Supporting Information: Coevaporation stabilizes tin based perovskites in a single Sn-oxidation state

Ajay Singh,[†] Jeremy Hieulle,[†] Joana Ferreira Machado,[†] Sevan Gharabeiki,[†] Weiwei Zuo,[‡] Muhammad Uzair Farooq,[†] Himanshu Phirke,[†] Michael Saliba,[‡,¶] and Alex Redinger[*,†]

†*Department of Physics and Materials Science, University of Luxembourg, Luxembourg City L-1511, Luxembourg*

‡*Institute for Photovoltaics (IPV), University of Stuttgart, Pfaffenwaldring 47, 70569 Stuttgart, Germany*

¶*Helmholtz Young Investigator Group FRONTRUNNER, IEK5-Photovoltaik, Forschungszentrum Jülich, 52425 Jülich, Germany*

E-mail: alex.redinger@uni.lu

Experimental section

To obtain the MASI films, CH_3NH_3I and SnI_2 were thermally evaporated in a vacuum chamber (at a base pressure of 2.0×10^{-7} mbar) enclosed in a glovebox. The partial pressure of SnI_2 and CH_3NH_3I were controlled by controlling their source temperatures. The SnI_2 source temperature was fixed at 265 °C. To obtain stoichiometric films, the CH_3NH_3I source temperature was varied between 110 °C to 128 °C to maintain a constant vapor pressure of 1×10^{-5} mbar during growth. To obtain SnI_2 rich films, the CH_3NH_3I source temperature

was varied between 106 °C to 115 °C. The deposition was set to 90 min to obtain $\simeq$ 300 nm perovskite films. The MASI samples are grown on ITO substrates, expect the sample used for thermal degradation investigation which is grown on glass.

To obtain Formamidinium Tin triiodide (FASI) film, 559 mg SnI_2, 236 mg Formamidinium iodide (FAI), 33.3 mg PEABr (phenyl ethyl ammonium), and 21.5 mg SnF_2 were dissolved in 1 ml of dimethyl sulfoxide (DMSO). The prepared precursor solution was then spin-coated onto the substrate at 4000 rpm for 70 s. Chlorobenzene was used as an anti-solvent during the spin-coating process and the films were annealed on a hot table at 100 °C for 40 min at the end of the spin-coating process.

X-ray diffraction was measured using a Bruker D8 Cu K_α diffractometer in θ-2θ Bragg-Brentano configuration. During the XRD measurements, the film is exposed to an X-ray beam of 40 mA, 40 kV with a beam diameter of 0.36 mm^{-2}. The XRD measurements were done in an N_2 atmosphere.

PL imaging measurements were done with a 532 nm pulsed laser (40 MHz repetition rate) for the degradation measurements under N_2. PL spectrum and quasi Fermi-level splitting measurements were done with a 660 nm CW-laser in air. Both system were independently calibrated to absolute photon numbers.

X-ray photoelectron spectroscopy (XPS) measurements were performed in an ultra-high vacuum (UHV) environment at a base pressure of 1-4$\times 10^{-10}$ mbar. A non-monochromatic Mg K_α source with a 1253.6 eV photon energy was used (power set to 300 W). The XPS spectra were recorded with a Prevac (Upper Silesia, Poland) analyzer, using a single channeltron. All the measurements were performed in the dark and the X-ray exposure of the fresh samples was limited to $<$30 min. The Sn 3d peak was used to track the oxidation states of tin in our perovskite materials and compared to a pure Sn plate taken as a reference.

The KPFM and AFM measurements were done both in ultra-high vacuum as well as in N_2 environments. The measurements in UHV allow for a better spatial resolution and a more accurate CPD measurement than in N_2 environments. The more precise measurements

are due to the higher quality factor of the cantilever in vacuum as respect to what would be obtained in atmospheric pressure. The use of a frequency modulation mode also allows to reduce artifacts and cross-talk between the KPFM and AFM signals. The AFM and KPFM maps presented in Figure 1 (main manuscript) were obtained in UHV in frequency modulation mode with a fixed excitation amplitude of 0.5V. The cantilever used has a Q factor of 38000 in UHV. For KPFM measurements an external lock-in amplifier was employed to measure the contact potential difference (CPD) between the tip and the sample. The typical lock-in parameters used are as follows: a modulation voltage of 0.4 V, a time constant of 1 ms, an excitation frequency of 965 Hz, and sensitivity of 5 ms.

The KPFM and AFM measurements were repeated under nitrogen environment to study topography and workfunction variations on a larger length scale compared to the UHV measurement. The relative humidity during the measurements was less than 8%, without exposing the samples to air. By combining KPFM with non-contact AFM, the topography and CPD measurements were acquired simultaneously over the same area of the sample. AFM measurements were conducted in the amplitude-modulation mode, while the KPFM signal was obtained in frequency modulation.

XRD of SnI_2 rich MASI film

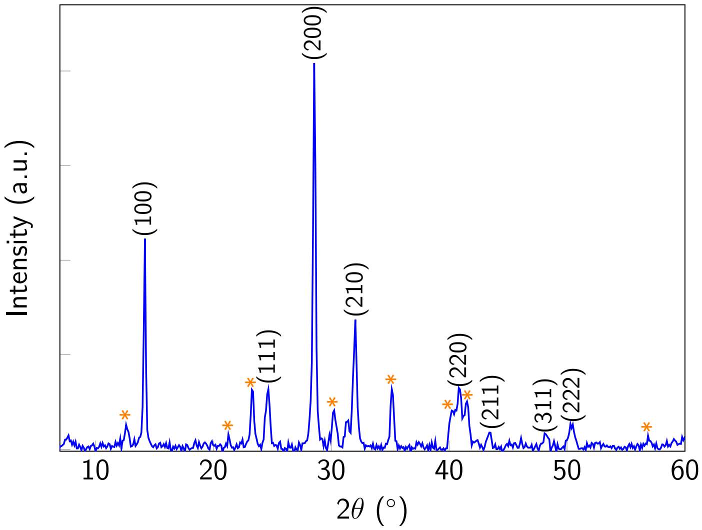

Figure S1: X-ray diffraction measurement for SnI_2 rich co-avaporated MASI film. * represents SnI_2 peaks,[1–3] confirming the excess SnI_2 in the deposited film.

SEM image of pristine MASI film

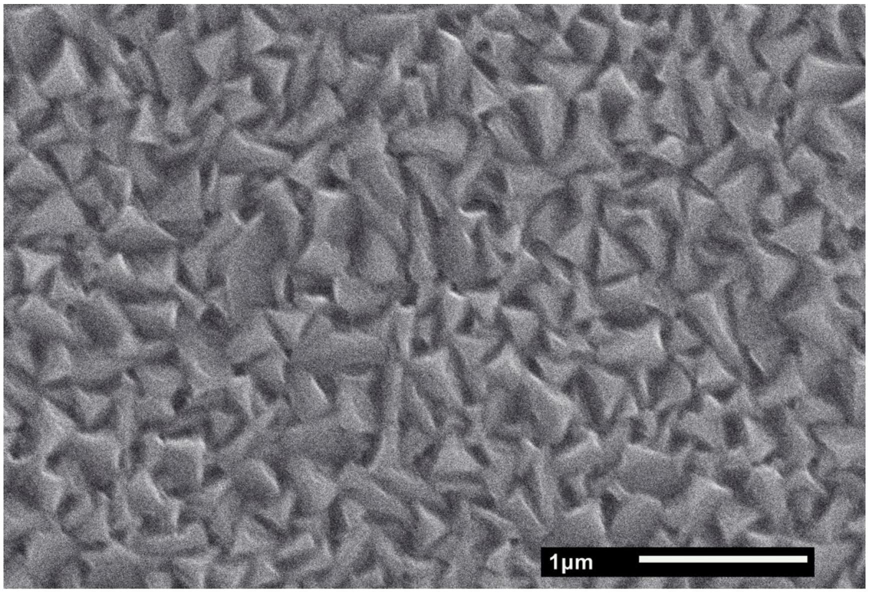

Figure S2: Top-view SEM image of PVD grown stoichiometric MASI perovskite film deposited on ITO/Glass.

Large area AFM/KPFM in N_2

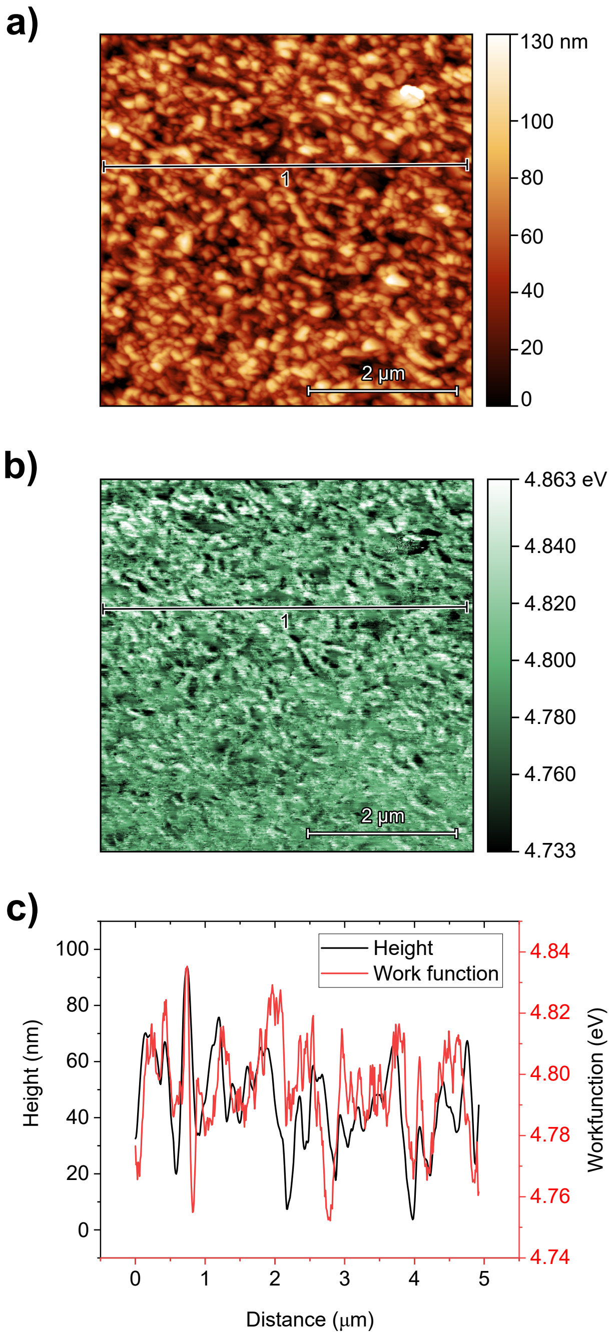

Figure S3: (a) Topography, and (b) surface workfunction map of the PVD-grown SnI_2 rich MASI film. The measurements are done in N_2 environment. (c) Line profiles extracted from (a): black curve, and (b): red line, respectively.

To scan a large area, we performed AFM (amplitude modulation mode) and KPFM

measurements in N_2. To ensure the uniformity and consistency of the results, the AFM scans were taken over several spots which were macroscopically away from each other. Figures S3(a) and S3(b) represent topography and workfunction maps recorded for SnI_2 rich MASI film. The measurement is done by using an AFM tip with an apex diameter of 20 nm. The resolution of the recorded images is therefore limited to 20 nm.[4] The MASI film exhibits a very smooth surface with a maximum height of 133.7 nm and an average roughness below 25 nm.

In the surface workfunction map, brighter regions represent the higher workfunction and vice versa. The average workfunction observed here is 4.79 eV covering almost 80% of the surface. The low variation in the workfunction indicates electronically uniform MASI film. Some of the small variations in the workfunction can be attributed to the facet-dependent contrast.[5] Studies have shown that the FM-KPFM technique is prone to tip artifacts.[4,6] To elucidate this phenomenon, line profiles that were simultaneously drawn at similar coordinates on both workfunction and topography map are shown in Figure S3(c). From here, we can infer that there is no direct correlation between the topography and workfunction. Moreover, we did not observe a secondary phase in the workfunction, maybe because the workfunction of $CH_3NH_3SnI_3$ and SnI_2 are similar.[7,8] Importantly, the non-stoicheometric films exhibit similar roughness and workfunction distribution as exhibited by the films not rich in SnI_2 (Refer to Figure 2 in the main manuscript).

PL degradation

For the Photoluminescence(PL) imaging based photodegradation study, a home-built PL setup is used. The setup consists of an InGaAs camera, capable of capturing images in energy range 0.9 eV to 1.4 eV, and a 532 nm pulsed green laser. The laser beam is homogenized to have a flat profile. The camera outputs a spatial map of the light-emitting surface of the illuminated region. The laser illuminates the sample periodically with a pulse rate of 20 MHz

to 40 MHz. The InGaAs camera captures the photoluminescence image of the sample every 2 min at a fixed integration time. Initially, the integration time is kept to 100 ms, which is later varied to capture significant photons from the photodegraded sample. The incident green laser energy fluence is set to 50 mW cm^{-2}. The reflected laser light is recorded as a reference to track change in the injection.

The photoluminescence quantum yield (Q_e^{lum}) is extracted from the recorded PL images. Figure S4a shows the Q_e^{lum} decay over time for non-stoichiometric MASI film. Interestingly, in the first few hours, the PL yield increases, in some cases even when the incident laser power goes down. This could be attributed to photo-soaking and photo-doping during the light exposure, similar to $CH_3NH_3PbI_3$ perovskite.[9–11] Importantly, the non-stoicheometric film shows stronger photo-doping in contrast to the film with no excess SnI_2.

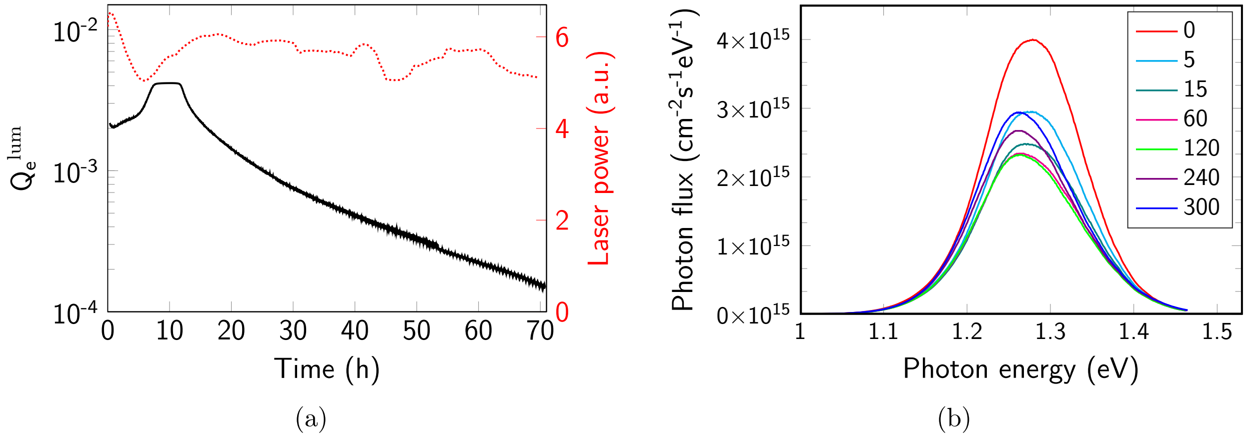

Figure S4: Photodegradation and photoluminescence spectrum characterization of non-stichiometric MASI film: (a) Measured photoluminescence quantum yield (Q_e^{lum}) over time, and (b) PL spectra recorded at different time intervals. Q_e^{lum} is extracted from the PL images recorded in N_2 environment while continuously illuminating the sample with green laser. Reflected laser light is measured as a reference for the injection. PL spectra are recorded in air, while continuously illuminating the sample with a red laser of 1 sun equivalent intensity.

For the non-stoichiometric MASI sample, a subset of recorded PL spectra is shown in Figure S4b. The measurements are done at 1 sun equivalent red laser light in air. For 0 min to 130 min, the PL intensity continuously decreases while illuminating the sample. Later, the PL intensity increases due to photo-soacking effect. From the recorded PL spectra, extracted

Q_e^{lum} is shown by the black curve in Figure S5. The change in the PL peak position is shown by the red curve in Figure S5.

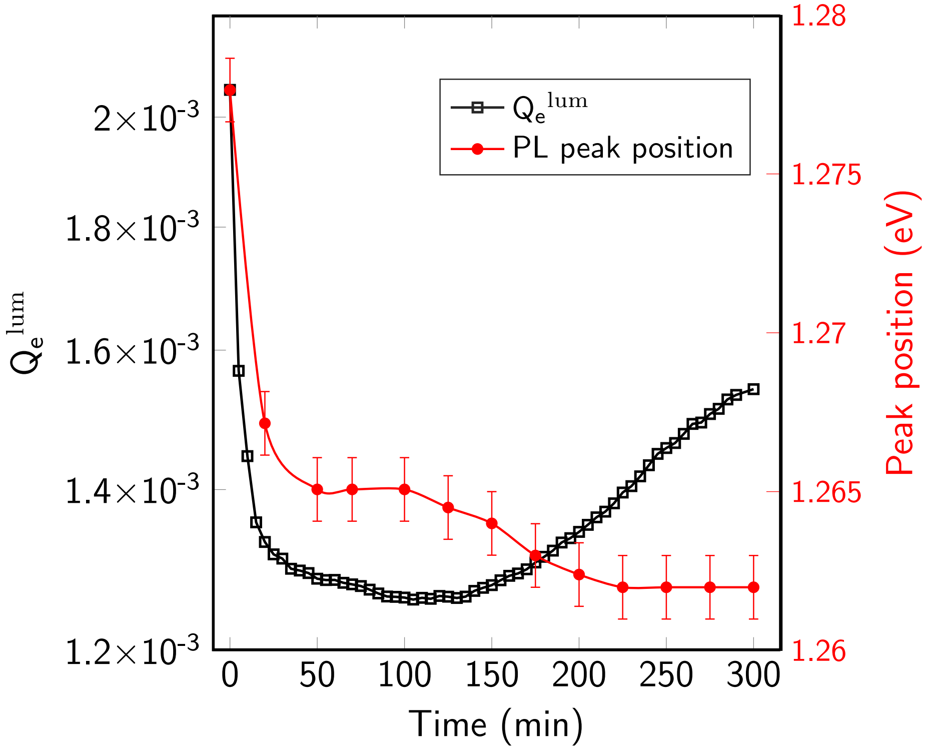

Figure S5: Measured PLQY and PL peak position for non-stoichiometric sample illuminated by a red laser at one sun equivalent in air.

Both the films with and without excess SnI_2 show a change in PL peak position and drop in Q_e^{lum} while exposed to red laser in air. However, the films behave differently upon light exposure in N_2 envirnoment. As mentioned earlier, the non-stoicheometric film exhibit much stronger photo-doping in in first few hours of light exposure under N_2. The photodegradation and the reason behind PL peak position are still not clear and dedicated investigation is needed.

References

(1) Kostko, V.; Kostko, O.; Makovetskii, G.; Yanushkevich, K. Thin film structure of tin (II) iodide. *Physica Status solidi (b)* **2002**, *229*, 1349–1352.

(2) Persson, K. Materials Data on SnI2 (SG:12) by Materials Project. 2014; doi.org/10.17188/1201402.

(3) Gates-Rector, S.; Blanton, T. The Powder Diffraction File: A Quality Materials Characterization Database. *Powder Diffr.* **2019**, *34*, 352–360, doi.org/10.1017/S0885715619000812.

(4) Nony, L.; Foster, A. S.; Bocquet, F.; Loppacher, C. Understanding the Atomic-Scale Contrast in Kelvin Probe Force Microscopy. *Phys. Rev. Lett.* **2009**, *103*, 036802.

(5) Kim, D.; Yun, J.-H.; Lyu, M.; Kim, J. C.; Lim, S.; Yun, J. S.; Wang, L.; Seidel, J. Probing Facet Dependent Surface Defects in $MAPbI_3$ Perovskite Single Crystals. *J. Phys. Chem. C* **2019**, *123*, 14144–14151.

(6) Lanzoni, E. M.; Gallet, T.; Spindler, C.; Ramírez, O.; Boumenou, C. K.; Siebentritt, S.; Redinger, A. The impact of Kelvin probe force microscopy operation modes and environment on grain boundary band bending in perovskite and $Cu(In,Ga)Se_2$ solar cells. *Nano Energy* **2021**, *88*, 106270.

(7) Marshall, K. P.; Walton, R. I.; Hatton, R. A. Tin perovskite/fullerene planar layer photovoltaics: improving the efficiency and stability of lead-free devices. *J. Mater. Chem. A* **2015**, *3*, 11631–11640.

(8) Ravindran, P.; Delin, A.; Ahuja, R.; Johansson, B.; Auluck, S.; Wills, J.; Eriksson, O. Optical properties of monoclinic SnI_2 from relativistic first-principles theory. *Phys. Rev. B* **1997**, *56*, 6851.

(9) Tian, Y.; Peter, M.; Unger, E.; Abdellah, M.; Zheng, K.; Pullerits, T.; Yartsev, A.; Sundström, V.; Scheblykin, I. G. Mechanistic insights into perovskite photoluminescence enhancement: light curing with oxygen can boost yield thousandfold. *Phys. Chem. Chem. Phys.* **2015**, *17*, 24978–24987.

(10) Galisteo-López, J. F.; Anaya, M.; Calvo, M.; Míguez, H. Environmental effects on the photophysics of organic–inorganic halide perovskites. *J. Phys. Chem. Lett.* **2015**, *6*, 2200–2205.

(11) Mosconi, E.; Meggiolaro, D.; Snaith, H. J.; Stranks, S. D.; De Angelis, F. Light-induced annihilation of Frenkel defects in organo-lead halide perovskites. *Energy Environ. Sci.* **2016**, *9*, 3180–3187.
